# Two Years of Evolutionary Dynamics of SARS-CoV-2 in Mexico, With Emphasis on the Variants of Concern

**DOI:** 10.3389/fmicb.2022.886585

**Published:** 2022-07-05

**Authors:** Alejandro Flores-Alanis, Gabriela Delgado, Luis F. Espinosa-Camacho, Flor Rodríguez-Gómez, Armando Cruz-Rangel, Luisa Sandner-Miranda, Alejandro Cravioto, Rosario Morales-Espinosa

**Affiliations:** ^1^Laboratorio de Genómica Bacteriana, Departamento de Microbiología y Parasitología, Facultad de Medicina, Universidad Nacional Autónoma de México, Mexico City, Mexico; ^2^Laboratorio de Análisis de la Biodiversidad y Genómica, Departamento de Bioingeniería Traslacional, Centro Universitario de Ciencias Exactas e Ingenierías, Universidad de Guadalajara, Guadalajara, Mexico; ^3^Laboratorio de Bioquímica de Enfermedades Crónicas, Instituto Nacional de Medicina Genómica, Mexico City, Mexico

**Keywords:** SARS-CoV-2, evolutionary dynamics, variants of concern, population dynamics, Mexico, Bayesian demographic analysis

## Abstract

**Background:**

The advance of the COVID-19 pandemic and spread of SARS-CoV-2 around the world has generated the emergence of new genomic variants. Those variants with possible clinical and therapeutic implications have been classified as variants of concern (VOCs) and variants of interest (VOIs).

**Objective:**

This study aims to describe the COVID-19 pandemic and build the evolutionary and demographic dynamics of SARS-CoV-2 populations in Mexico, with emphasis on VOCs.

**Methods:**

30,645 complete genomes of SARS-CoV-2 from Mexico were obtained from GISAID databases up to January 25, 2022. A lineage assignment and phylogenetic analysis was completed, and demographic history for Alpha, Gamma, Delta and Omicron VOCs, and the Mexican variant (B.1.1.519) was performed.

**Results:**

148 variants were detected among the 30,645 genomes analyzed with the Delta variant being the most prevalent in the country, representing 49.7% of all genomes.

**Conclusion:**

The COVID-19 pandemic in Mexico was caused by several introductions of SARS-CoV-2, mainly from the United States of America and Europe, followed by local transmission. Regional molecular epidemiological surveillance must implement to detect emergence, introductions and spread of new variants with biologically important mutations.

## Introduction

The first outbreak of coronavirus disease 2019 (COVID-19) by the severe acute respiratory syndrome coronavirus 2 (SARS-CoV-2) was reported in the city of Wuhan, Hubei province, China in late December 2019. Since then, SARS-CoV-2 has been spreading throughout the world presenting different mutations and developing new genomic variants of the virus, which are circulating globally.^1^ Mutation is an inherent characteristic of all viruses; however, the fate of most of the new mutations is regulated by natural selection and genetic drift; therefore, the frequency of mutations can randomly increase, decrease, disappear, or persist (Moya et al., 2004; Davies et al., 2021). To date, several variants of SARS-CoV-2 have been detected around the world, but only a small number of them present a public health risk. Three scientific nomenclature systems have been used to identify and track SARS-CoV-2 genotypes, but on May 31, 2020, the Virus Evolution Working Group of the World Health Organization (WHO) announced its recommendations for a new nomenclature system for SARS-CoV-2 variants of concern (VOCs; Parums, 2021). The VOCs are those strains that show characteristics, such as higher transmissibility, and are associated with more severe disease, but the immune response generated by previous infection or vaccination is poor for the control of these VOCs. Until January 25, 2022, the VOCs circulating around the world have been Alpha (lineage B.1.1.7 and eight sub-lineages), Beta (lineage B.1.351 and five sub-lineages), Gamma (lineage P.1 and 22 sub-lineages), Delta (lineage B.1.617.2 and 215 sub-lineages) and Omicron (B.1.1.529 and three sub-lineages), which emerged independently in different regions (see footnote 1).

The first COVID-19 case confirmed in Mexico was reported on February 28, 2020, in Mexico City. The same day, a Phase 1 strategy was launched, which consisted of implementing specific measures to prevent the spread of the virus in the Mexican population. During Phase 1, most of the reported COVID-19 cases in Mexico were identified as imported cases. On March 24, 2020, the Mexican Health Ministry declared a Phase 2 strategy after detecting the first locally generated cases. The Mexican Health Ministry implemented voluntary isolation, curtailed all non-essential activities, and closed schools, government offices and shopping centers. Additionally, public health measures were implemented to prevent viral spread, such as the use of face masks, frequent hand washing and disinfection of public areas. However, these prevention measures were not sufficient, and due to a rapid increase in the number of cases and deaths by COVID-19, a national health emergency was declared in Mexico on March 30, 2020. On April 21, 2020, Phase 3 of the prevention strategy was initiated, which suspended almost all private and social activities (Suárez et al., 2020). Despite these strategic measures to prevent the spread of SARS-CoV-2, the number of cases and deaths continued to increase. To date, four COVID-19 waves have been observed in Mexico, the first from May to September 2020, the second from November 2020 to February 2021, which coincided with the emergence of the Mexican variant (B.1.1.519; Barona-Gómez et al., 2021), the third from July to September 2021, which coincided with the occurrence of the Delta VOC, and the last wave started in the first days of January 2022 with the introduction of the Omicron VOC. As on January 23, 2022, Mexico has reported a total of 4,667,829 COVID-19 cases and 303,183 deaths.^2^ Our study describes the population dynamics of SARS-CoV-2 throughout all 32 states of the Mexican territory and analyzes the mutational evolution of SARS-CoV-2 strains throughout the pandemic, with emphasis on the emergence of VOCs.

## Materials and Methods

### Sequences and Alignments

A total of 28,297 complete genomes of SARS-CoV-2 from Mexico were obtained from GISAID databases^3^ that had been reported up to December 1, 2021, using the following filters: complete (genomes>29,000 nucleotides), high coverage (<1% undefined bases (Ns) and < 0.05 unique amino acid mutations (not seen in other sequences in database) and no insertion/deletion unless verified by submitters) and complete collection date. To analyze the recent emergence of the Omicron VOC we made a second cohort from the GISAID database (see footnote 3) up to January 25, 2022, using the filters: variant Omicron, complete and complete collection date. In this second cohort, 2,348 genomes of the Omicron variant were recovered (Supplementary Table S1). All genomes were aligned using MAFFT v7.3 (Katoh and Toh, 2010) and revised by the BioEdit v7.2 software (Hall, 1999). The isolate from Wuhan, Hu-1 (GenBank: NC045512) was used as the reference genome. The non-coding 5′ and 3′ regions were trimmed off.

### Lineage Assigner and Phylogenetic Analysis

Lineage assessment was obtained using the Phylogenetic Assignment of Named Global Outbreak LINeages (PANGO LINeages) tool.^4^ To determine the origins and introduction of the SARS-CoV-2 and VOCs into Mexico, we constructed a phylogenetic tree of the genomes reported in Mexico during the start of the pandemic (February to March, 2020). In addition, two different phylogenetic trees were constructed for each Alpha, Beta, Delta, Gamma, and Omicron VOCs, one using the first genomes reported at the beginning of each outbreak and another for the total genomes of each VOC. Moreover, the Mexican variant (B.1.1.519), which represented 21.02% of the complete genomes analyzed, was included in the analyses. Due the great number of genomes reported for Delta VOC, we used a representative sample of 5,600 genomes. Maximum Likelihood phylogenetic trees were constructed using the Nextstrain platform.^5^ This tool provided information to differentiate between a group of sequences selected by the Nextstrain platform (which were used as reference sequences) and our Mexican SARS-CoV-2 genomes. For tree exploration, Auspice v0.6.0^6^ by Nextstrain was used. The trees were visualized and edited using the FigTree v1.4.3.^7^

### Bayesian Demographic Analyses

The analyses were performed for Alpha, Gamma and Delta VOCs, and the Mexican variant (B.1.1.519). A representative sample of 190 genomes of each variant was used for the analyses. Genomes that present non-determined (N) or other ambiguous nucleotides according to the IUPAC nucleotide code were excluded from the analysis. The Beta and Omicron VOCs were discarded due them presenting low-quality genomes. The dynamics of the demographic population were estimated using a Bayesian Skyline Plot (BSP) method performed in BEAST 2 v2.5.0 (Bouckaert et al., 2014) using the HKY + I substitution model (Duchene et al., 2020). Each dataset was analyzed using an uncorrelated log normal clock model with an evolution rate of 8.0 × 10^−4^ substitutions/site/year (1.4 × 10^−4^ to 1.31 × 10^−3^).^8^ For each analysis, one Markov Chain Monte Carlo (MCMC) was run for 200 million steps; the trees were sampled every 100,000 generations. Convergence was assessed based on effective sample size (ESS) using Tracer v1.5;^9^ only ESS values >200 were accepted. Uncertainty in the estimates was indicated by 95% highest posterior density (HPD) and the BSP was obtained by adjusting the age of the youngest tip.

## Results

### The Beginning of the Pandemic in Mexico

**Figure 1:**
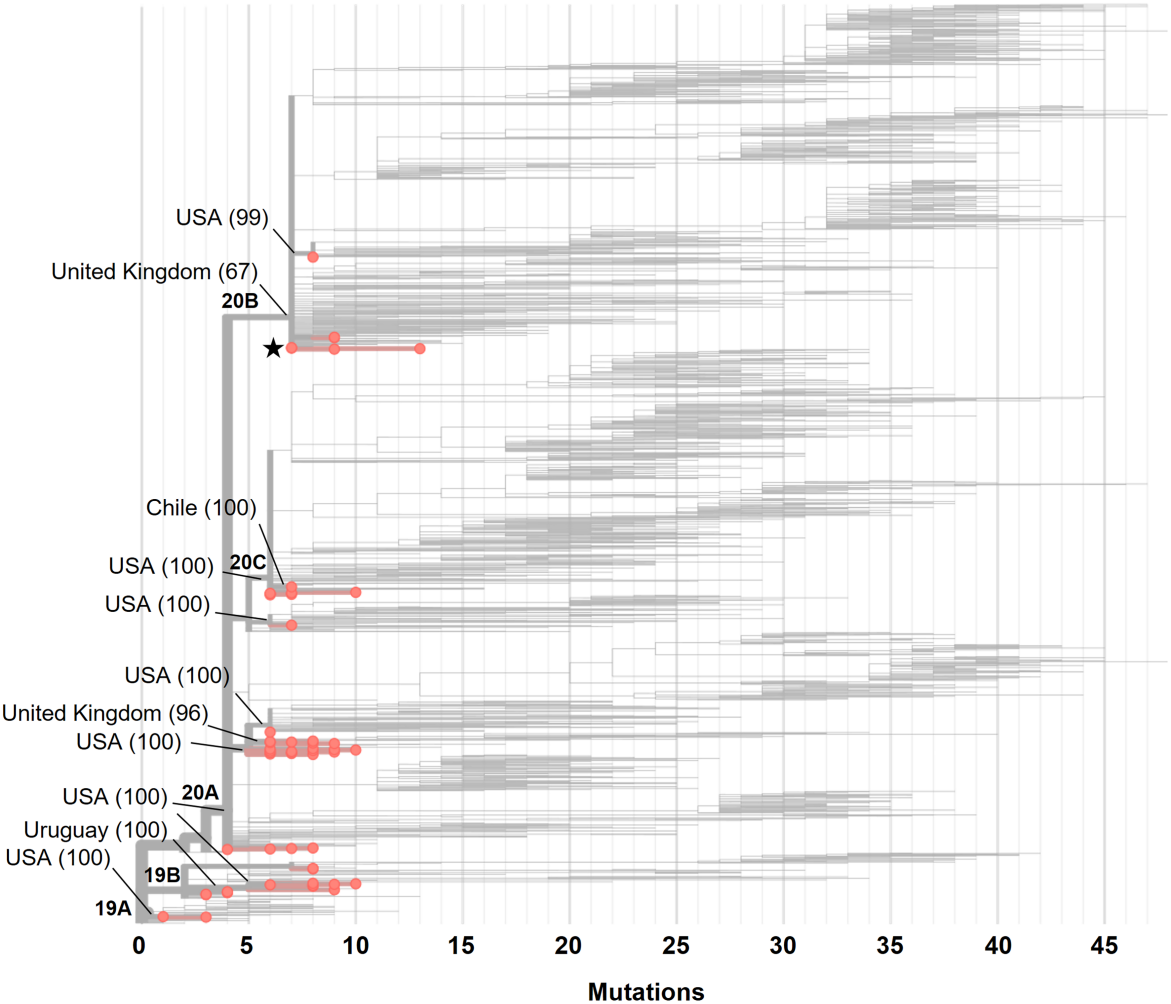
A Maximum-likelihood phylogeny of 56 SARS-CoV-2 genomes isolated in Mexico from February to March 2020. The branches with red circles represent the 56 genomes analyzed, and branches without a circle represent the 1,794 genomes that the Nextstrain took as reference. The principal clades (19A, 19B, 20A, 20B and 20C) are in the base of the nodes. The black star indicates the two isolates from February 2020. The lines indicate the introductions events from different regions and in parenthesis the confidence % value. USA, United States of America.

The phylogenetic analysis of 56 genomes available at the beginning of the pandemic (February and March 2020) showed that there were at least 12 importation events of SARS-CoV-2 into Mexico (Figure 1). We detected two importations from Europe (United Kingdom), two from South America (Chile and Uruguay) and the rest were from the United States of America (United States). In general, we found 13 variants, with the most frequent being B.1 (33.9%, *n* = 19), B.1.609 (16.1%, *n* = 9) and B.1.1 (12.5%, *n* = 7). The two samples isolated from Mexico City in February 2020 belonged to variant B.1.1, derived from a European variant. By April 2020, the number and frequency of the variants changed, and although we found 17 variants, the same variants were still the most frequent: B.1 (40%, *n* = 140) and B.1.609 (20.29%, *n* = 71), followed by variant B.1.243 (10.84%, *n* = 44).

### SARS-CoV-2 Variants in Mexico

**Figure 2:**
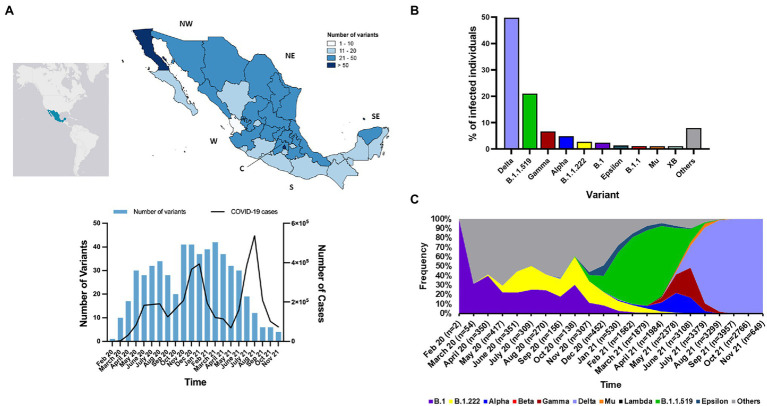
Geographic and temporal distributions of SARS-CoV-2 variants in Mexico. **(A)** Geographical distribution of the number of SARS-CoV-2 variants, temporal changes of the number of SARS-CoV-2 variants and monthly incidence of COVID-19 cases in Mexico. NW, northwest; NE, northeast; W, west; C, central; S, south; SE, southeast. **(B)** Frequencies of the most prevalent variants detected in Mexico. The variants with frequencies <1.0. are indicated in gray **(C)** Temporal frequency of the Alpha, Beta, Gamma and Delta VOCs, and other variants of interest. Jan, January; Feb, February; Aug, August; Sep, September, Oct, October; Nov, November; Dec, December.

We analyzed of the first cohort, which comprised of 28,297 genomes between February 2020 and November 2021. The second cohort that included only genomes of Omicron VOC was analyzed independently. According to the PANGO LINeages analysis, a total of 147 SARS-CoV-2 variants were detected among the 28,297 genomes from Mexico. The number of variants by state in the country ranged from 10 to 82. The state with the greatest number of variants was Mexico City in the central (C) region of Mexico, while the state with the lowest number of variants was Nayarit on the west (W) region (Figure 2A). In 2020, the number of variants increased with some fluctuations similar to the number of COVID-19 cases, but in 2021 incongruences between numbers of variants and COVID-19 cases were observed (Figure 2A). Throughout 2020, the diversity of variants was higher than in 2021, 103 and 81, respectively. In general, between February 2020 and November 2021, the frequency of variants ranged between 0.003 and 49.70% (Figure 2B). The most prevalent variants were Delta VOC and the Mexican variant (B.1.1.519); they represented 70.72% of all the genomes analyzed. The most prevalent variants in 2020 were B.1 and B.1.222, which together represented 37.74% of the genomes from that year. However, in 2021, the most prevalent variants were the Mexican variant in early 2021 and Delta VOC in mid-late 2021, which together represented 78.12% of the genomes analyzed in that year (Figure 2C). The breakdown of number of genomes identified as VOCs and other variants of interest as in Figure 2C is showed in Supplementary Figure S1.

### Evolutionary Dynamics of the SARS-CoV-2 VOCs in Mexico

Of all genomes analyzed, 61.24% (*n* = 17,329) were identified as VOCs as follows: Alpha (4.84%), Beta (0.05%), Gamma (6.64%) and Delta (49.70%) variants. Meanwhile, only 1.30% (*n* = 368) were identified as Lambda (0.21%) and Mu (1.09%) variants of interest.

#### Alpha VOC

**Figure 3:**
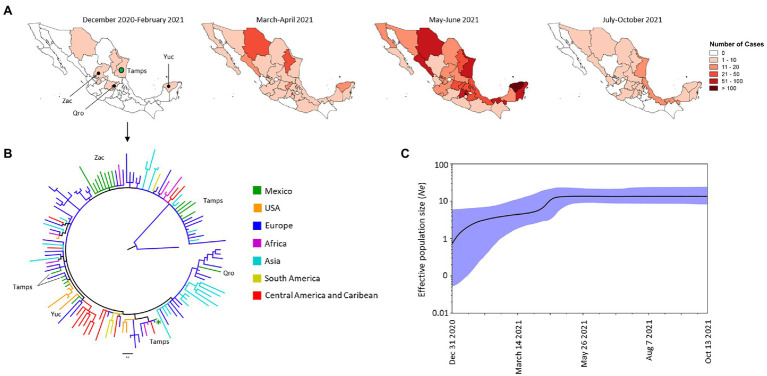
Spatial distribution and phylodynamics of the Alpha VOC in Mexico. **(A)** Temporal distribution of the Alpha VOC in Mexico. The green circle indicates the Mexican state of Tamaulipas (Tamps) where the Alpha VOC was first detected. Zac, Zacatecas; Qro, Queretaro; Yuc, Yucatan. **(B)** Phylogenetic analysis of Alpha VOC. The green branches indicate the 21 genomes of the Alpha VOC isolated in Mexico between December 2020 and February 2021; the isolates of the same VOC taken from other parts of the world that the Nextstrain took as references are shown in other colors. The green asterisks show the firs genome of Alpha VOC detected in Mexico. USA, United States of America. **(C)** Bayesian Skyline Plot of 190 genomes of the Alpha VOC. X axis shows the time and Y axis shows the effective population size (*Ne*). The middle line indicates the median estimates of the *Ne*, and the blue shaded area represents 95% highest posterior densities (HPD). Dec, December; Aug, August; Oct, October.

Among all the VOCs identified in Mexico during the period of our study, the Alpha variant was the third most frequent (Figure 2B); the first sequenced genome of an Alpha VOC in Mexico was extracted from an isolate from Tamaulipas (Tamps) in December 2020 (Figure 3A). The phylogenetic tree suggested that between December 2020 and February 2021 there were at least seven introduction events of Alpha VOC in four states of Mexico: Tamaulipas to the northeast (NE), Zacatecas (Zac) to the northwest (NW), Queretaro (Qro) in the central region and Yucatan (Yuc) to the southeast (SE; Figures 3A,B). These isolates were related to viruses from Europe (Romania, Greece, Netherlands, Denmark, Czech Republic, Ireland, Norway, England, Sweden, Albania, Moldova, Ukraine, and Montenegro), Africa (Coted-Ivoire, Central African Republic, Somalia, and Morocco), Asia (Kazakhstan and Pakistan), the Caribbean (Curacao) and the United States (Figure 3B). The first genome dated December 31, 2020, was closely related to a genome from Europe (Netherlands and Montenegro) and to others from the Caribbean (Curacao) and Africa (Coted-Ivoire and Morocco). Over time, comb-like structures in the phylogenetic tree of the Mexican isolates were formed (Supplementary Figure S2); the total of Mexican genomes of the Alpha VOC were closely related to genomes from different regions, but mainly to genomes from Europe and the United States.

From January to February 2021, the Alpha VOC spread to other states in the NW, NE, and W part of Mexico. It spread rapidly to other states and during March and April 2021 was detected in 27 states, and by June 2021, it had spread to almost all the states of Mexico. However, its frequency and reach was reduced drastically between July and October 2021 (Figure 3A), and the last isolate of this variant was collected in October 13, 2021. In terms of the effective population size (*Ne*) of the Alpha VOC, the BSP showed a constant increase between January and April 2021, when the increase slowed reaching a stationary phase that continued until October 2021 (Figure 3C).

#### Gamma VOC

**Figure 4:**
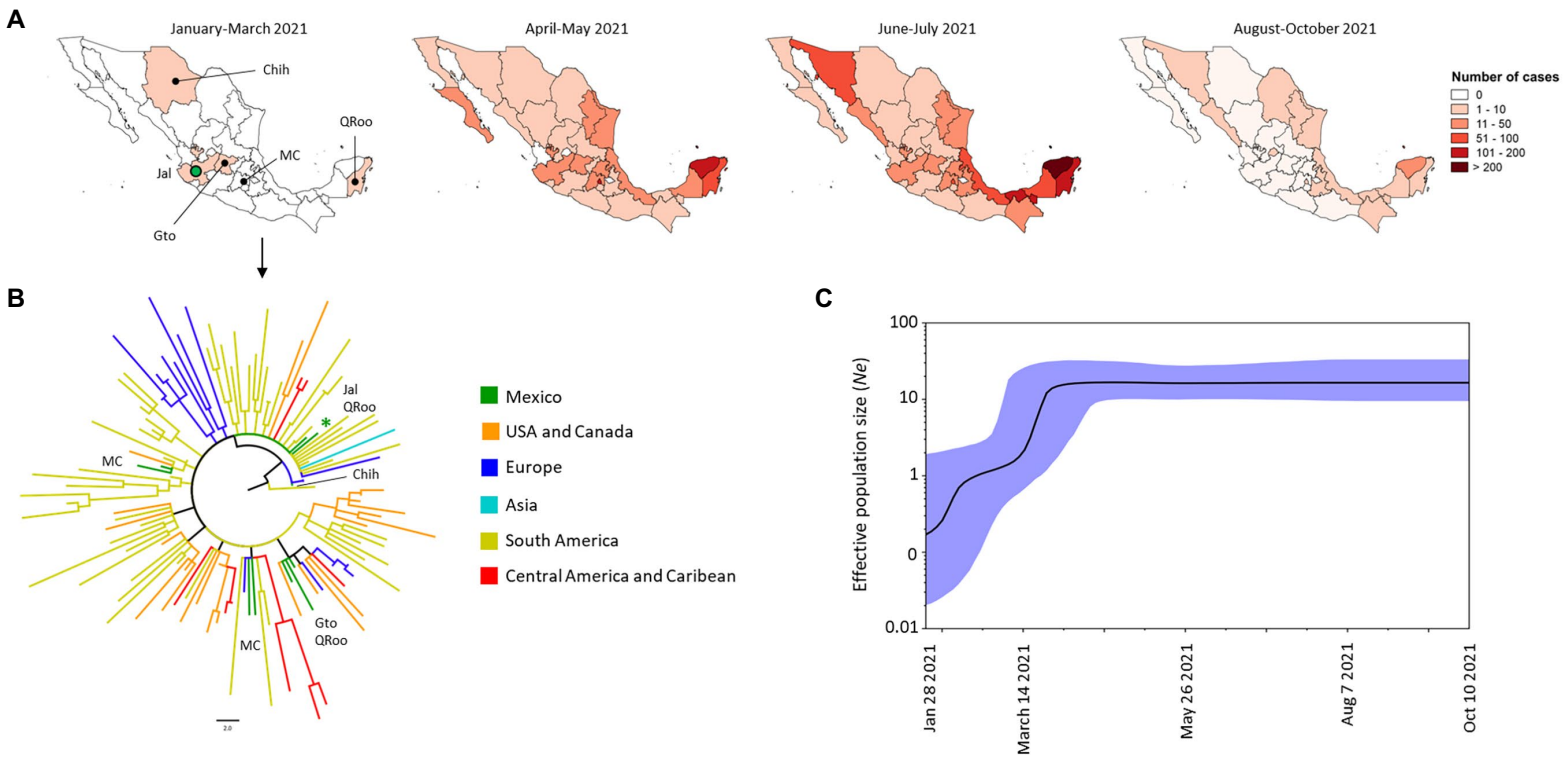
Spatial distribution and phylodynamics of the Gamma VOC in Mexico. **(A)** Temporal distribution of the Gamma VOC into Mexico. The green circle indicates the Mexican state of Jalisco (Jal) where the Gamma VOC was first detected. Chih, Chihuahua; Gto, Guanajuato; MC, Mexico City, QRoo, Quinta Roo. **(B)** Phylogenetic analysis of Gamma VOC. The green branches indicate the 10 genomes of the Gamma VOC isolated in Mexico between January and March 2021; the isolates of the same VOC taken from other parts of the world that the Nextstrain took as references are shown in other colors. The green asterisk indicates the genome from January 2021. USA, United States of America. **(C)** Bayesian Skyline Plot of 190 genomes of the Gamma VOC. X axis shows the time and Y axis shows the effective population size (*Ne*). The middle line indicates the median estimates of the *Ne* and the blue shaded area represents 95% highest posterior densities (HPD). Jan, January; Aug, August; Oct, October.

The Gamma VOC was the second most frequent VOC in Mexico and its first sequenced genome in Mexico was obtained from the state of Jalisco (Jal) in January 2021 (Figure 4A). The phylogenetic tree suggests that this VOC was introduced to the western states of Jalisco and Guanajuato (Gto), Mexico City (MC) in the center of the country, Chihuahua (Chih) in the NW, and Quintana Roo (QRoo) in the SE of Mexico. The first sequenced genomes of this VOC in Mexico were closely related to isolates from South America (Brazil, Uruguay, Suriname, and Peru), Europe (France) and Asia (Jordan). The isolates from Mexico City were closely related to isolates from Europe (Spain), South America (Brazil and Colombia) and the United States. The isolates from Chihuahua were closely related to isolates from Europe (Belgium), those from Guanajuato were closely related to isolates from the United States, while those from Quintana Roo were like the isolates from Jalisco and Guanajuato (Figure 4B). Over time, monophyletic clades, and comb-like structures in the phylogenetic tree of the Mexican isolates were observed (Supplementary Figure S2); in general, the isolates of this VOC from Mexico were closely related to isolates from South America, Europe, and the United States.

From April to May 2021, the frequency of this VOC increased (Figure 2C) and was present in 29 states (Figure 4A). By July 2021, only the state of Tlaxcala in the center of Mexico was free of cases caused by the Gamma VOC, while the SE of the country presented the highest number of cases (Figure 4A). But, between August and October 2021, the number of cases by this VOC decreased drastically (Figure 2C) and was only detected in 14 states of Mexico at low levels of frequency (Figure 4A). The demographic dynamics of the Gamma VOC was similar to that of the Alpha VOC, with a stationary phase from April to October 2021 (Figure 4C).

#### Delta VOC

**Figure 5:**
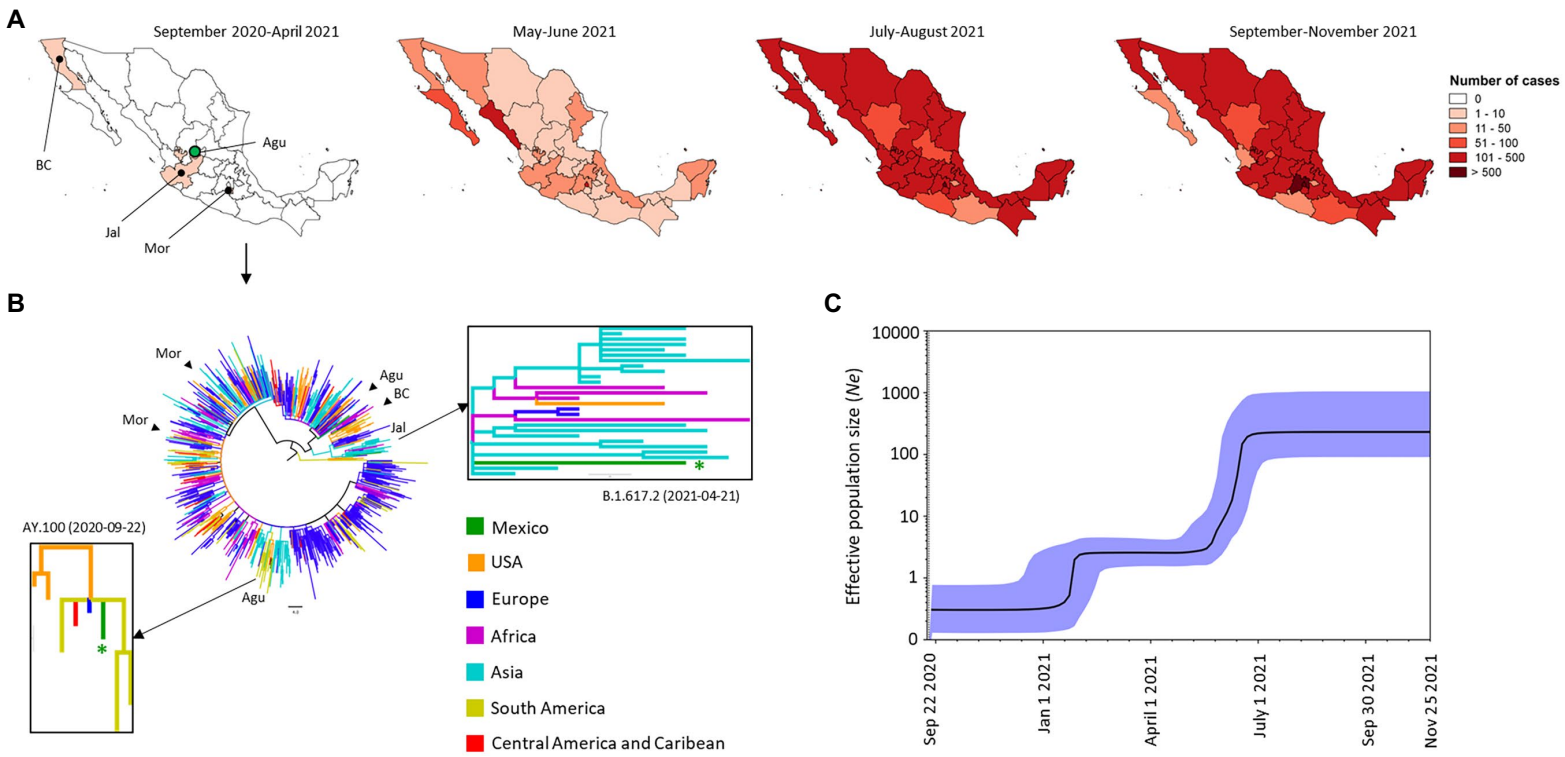
Spatial distribution and phylodynamics of the Delta VOC in Mexico. **(A)** Temporal distribution of the Delta VOC in Mexico. The green circle indicates the Mexican state of Aguascalientes (Agu) where Delta VOC was first detected. BC, Baja California; Jal, Jalisco; Mor, Morelos. **(B)** Phylogenetic analysis of Delta VOC. The green branches indicate the 6 genomes of the Delta VOC isolated in Mexico between September 2020 and April 2021; the isolates of the same VOC taken from other parts of the world that the Nextstrain took as references are shown in other colors. The introduction events of the first genome of the lineage B.1.617.2 and sub-linage AY.100 are indicated by a closeup of the tree branches. The arrowheads indicate the position of the others introduction events of the Delta VOC. USA, United States of America. **(C)** Bayesian Skyline Plot of 190 genomes of Delta VOC. X axis shows the time and Y axis shows the effective population size (*Ne*). The middle line indicates the median estimates of the *Ne* and the blue shaded area represents 95% highest posterior densities (HPD). Sep, September; Jan, January; Nov, November.

Delta was the most frequent VOC in Mexico (Figure 2B) with the first genome being reported in Mexico in September 2020 in the state of Aguascalientes (Agu). Following the initial identification of the Delta VOC, five more genomes were reported between January and April 2021. They were found in the states of Aguascalientes (*n* = 1) and Jalisco (Jal; *n* = 1) in the W, Morelos (Mor; *n* = 2) to the south, and Baja California (BC; *n* = 1) in the NW of Mexico (Figure 5A). The phylogenetic tree suggests that these cases were imported independently. The first sequenced genome was isolated in Aguascalientes as the sub-lineage AY.100 imported from the United States (Figure 5B). The genomes from Morelos and Aguascalientes detected in January 2021 were closely related to isolates from Europe (Czech Republic and Italy) and Asia (Hong Kong and Philippines), while the isolated from Baja California detected in February 2021 was close related to isolates from Europe (Czech Republic), the United States, and Canada. Those four genomes were identified as the sub-lineages AY.20, AY.26 and AY.13. Meanwhile, the isolate sequenced in April 2021 in Jalisco was identified as lineage B.1.617.2 and was related to isolates from Asia (Hong Kong and Oman; Figure 5B). Monophyletic clades and comb-like structures in the phylogenetic tree of the Mexican isolates were observed over time (Supplementary Figure S2). The genomes of this VOC were closely related to genomes from various regions, principally to genomes from the United States.

From May to June 2021, the Delta VOC increased its frequency rapidly displacing Alpha and Gamma VOCs (Figure 2C); at this point it spread to 31 states in Mexico (Figure 5A). By August 2021, the entire Mexican territory presented this VOC in high frequency. This situation prevailed in the country between September and November 2021, with Mexico City and the State of Mexico being the most affected (Figure 5A). The Delta VOC represented 99.75% (*n* = 7,354) of the genomes analyzed in that period (Figure 2C). Of the 215 sub-lineages reported around the world, 72 were identified in Mexico, the most prevalent were AY.20 (44.6%, *n* = 6,278), AY.26 (27.2%, *n* = 3,835) and AY.100 (8.5%, *n* = 1,196). The BSP showed that the *Ne* of Delta VOC increased over time. It presented a stationary phase after its introduction into Mexico and increased in late January 2021, followed by a second stationary phase and then a drastic increase again between May and June 2021. The *Ne* became constant in November 2021 (Figure 5C).

#### Beta VOC

The Beta variant was the least frequent VOC in Mexico. The phylogenetic tree suggests that this VOC was imported to Mexico in three independent events; the first event was to the state of Campeche (Camp) in the SE of Mexico in April 2021, after this, nine more cases were documented in Campeche, one in Tabasco (Tab) to the SE, and one in Tamaulipas (Tamps) to the NE of Mexico (April–May 2021). The second introduction event was into the state of Nuevo Leon (NL) to the NE of Mexico in April 2021, with an additional case being reported in the same state in May 2021. The last introduction was into Baja California (BC) in the NW of Mexico in April 2021 (Supplementary Figures S3A,B). The Mexican viruses were closely related to isolates from Europe (Spain, Wales, and Luxemburg), Africa (South Africa, Reunion, and Djibouti), and Canada (Supplementary Figure S3B).

#### Omicron VOC

**Figure 6:**
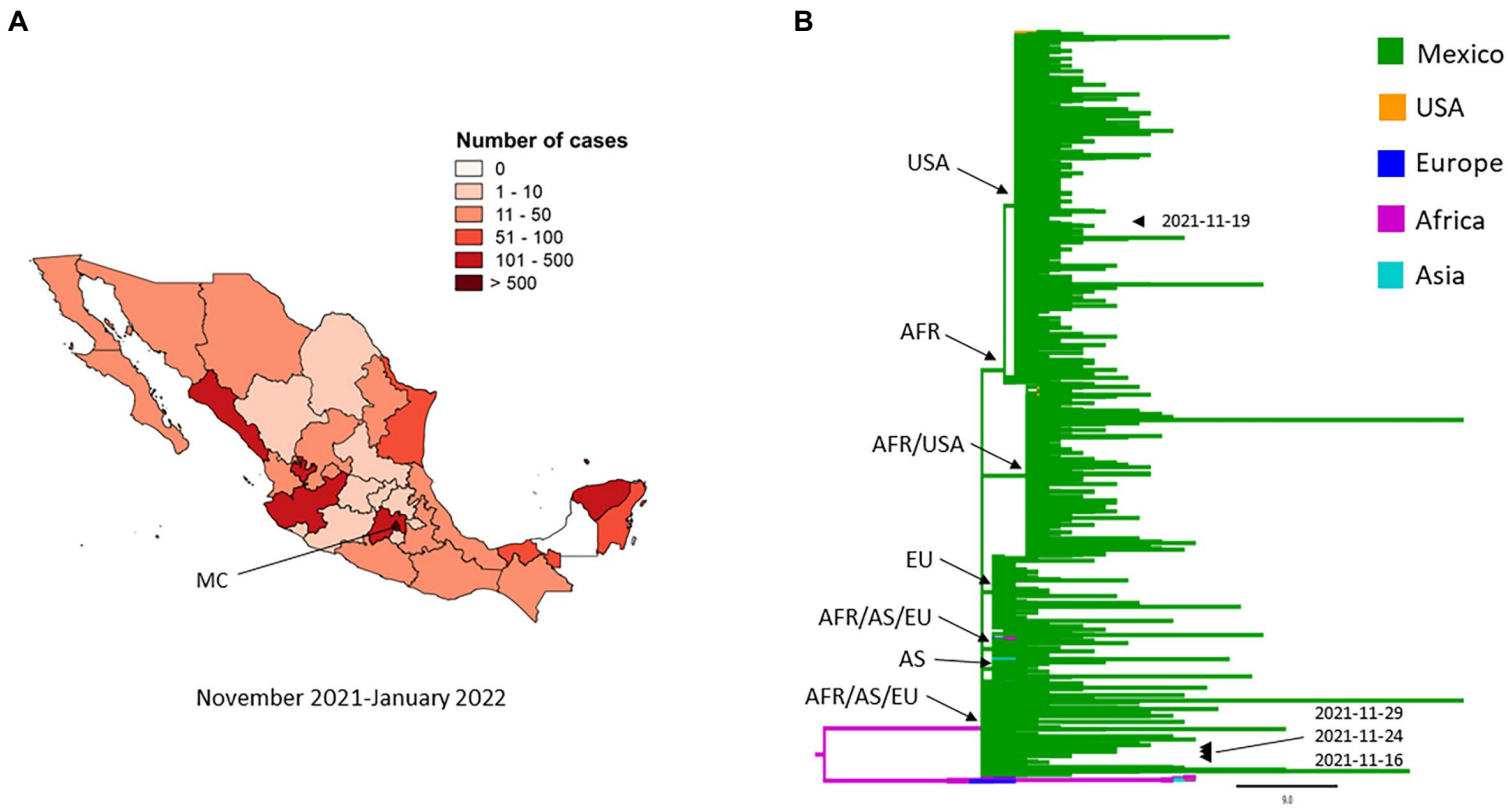
Spatial distribution and phylodynamics of Omicron VOC in Mexico. **(A)** Geographical distribution of the Omicron VOC in Mexico. MC, Mexico City. **(B)** Phylogenetic analysis of Omicron VOC. The green branches indicate the 2,348 genomes of the Omicron VOC isolated in Mexico. The isolates of Omicron VOCs taken from other parts of the world that the Nextstrain took as references are shown in other colors. The arrowheads indicate the 4 genomes from November 2021 and the arrows indicate the formation of the comb-like structures shared with isolates from different regions. USA, United States of America; AFR, Africa, EU, Europe; AS, Asia.

The first genome of the Omicron variant identified in Mexico was dated November 16, 2021; the sample was collected in Mexico City (MC; Figure 6A), and in the same month, three more cases were reported in the same city, two cases were similar to the genome from November 16, 2021, and the third case was a new introduction (Figure 6B). By January 2022, the number of infections and spread of this VOC increased drastically with Mexico City being the most affected (Figure 6A). The phylogenetic tree resolved seven comb-like structures, each of which were represented by isolates from Mexico shared with isolates from the United States, Africa (Ghana and South Africa), Europe (Portugal, United Kingdom, Belgium, and Denmark), South America (Brazil), and Asia (Singapore, Hong Kong, Japan, and Australia; Figure 6B). Of the four genomes sequenced from samples taken in November 2021, one was closely related to isolates from the United States and three to isolates from Africa, Asia, and Europe (Figure 6B).

### Other Variants: Evolutionary Dynamics of the Mexican Variant (B.1.1.519) of SARS-CoV-2 in Mexico

**Figure 7:**
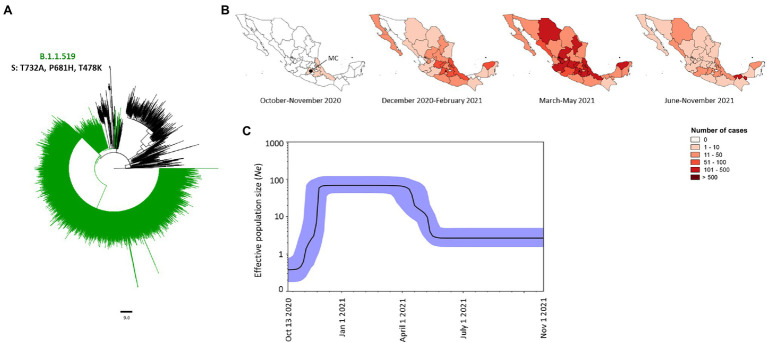
Phylogeny, frequency, and demographic dynamics of the Mexican variant (B.1.1.519) in Mexico. **(A)** Phylogenetic tree of 5,949 SARS-CoV-2 isolates from Mexico. The Mexican variant harboring mutations T732A, P618H and T478K in the Spike protein is in green. The isolates that the Nextstrain took as references are shown in black. **(B)** Temporal distribution of the Mexican variant. MC, Mexico City. **(C)** Bayesian Skyline Plot of 190 genomes of the Mexican variant, X axis shows the time and Y axis shows the effective population size (*Ne*). The middle line indicates the median estimates of the *Ne* and the blue shaded area represents 95% highest posterior density (HPD) interval. Jan, January; Oct, October; Nov, November.

The Mexican variant was the second variant most prevalent in Mexico representing 21.02% (*n* = 5,949) of all genomes analyzed (Figure 2B). This variant was characterized by three mutations in the Spike protein, namely T732A, P681H and T478K (Figure 7A). This variant was first detected in Mexico City (MC) in October 2020, and it spread to neighboring states, such as the State of Mexico in the center, and Puebla and Veracruz to the south of Mexico (Figure 7B). Between December 2020 and February 2021, it increased its frequency and was detected in 29 states of Mexico (Figure 7B) displacing other variants, such as B.1 and B.1.1.222 (Figure 2C). Between March and May 2021, this variant reached its highest peak of cases, representing 64.2% (*n* = 4,008) of the genomes analyzed in that period (Figure 2C). By that time, this variant was presented in the whole of Mexico with Mexico City being the most affected (Figure 7B). Between June and November 2021, the number of cases decreased dramatically with 30 states reporting cases in low frequency (Figure 7B). The last isolate of this variant was reported on November 1, 2021. The BPS showed that the dynamic of the *Ne* of this variant was similar to its rate of frequency with the *Ne* increasing by November 2020 and reaching a stationary phase between December 2020 and March 2021, after which its *Ne* started to decrease between April 2021 and middle-May and a second stationary phase remained constant until November 2021 (Figure 7C).

## Discussion

Although the number of genomes sequenced in Mexico is not proportional to the burden of COVID-19 infection, we analyzed a representative number of genomes reported up to January 25, 2022. Our objective was to study the evolutionary and demographic dynamics of the SARS-CoV-2 in Mexico with emphasis on variants of concern (VOCs).

After the emergence of SARS-CoV-2 in Wuhan, China in December 2019, the virus began to spread to other countries of the world, accumulating mutations that had derived in different variants. In Mexico, the first case of COVID-19 was reported on February 28, 2020, in a 35-year-old adult who arrived from Bergamo, Italy.^10^ After sequencing and analyzing this isolate, it turned out to be variant B.1.1, which was the second most frequent variant in the north of Italy at the time (Giovanetti et al., 2021). Epidemiological data from the Mexican Health Ministry indicated that the first COVID-19 cases (3.88%, *n* = 746) were imported from the United States of America (USA), Canada, Europe (Italy, Germany, Spain, France, the United Kingdom, Switzerland and Czech Republic), the Middle East (Turkey), South America (Peru, Colombia, Brazil and Argentina), and Asia (China and Singapore); 0.7% (*n* = 135) of the cases were reported as the result of contact with imported cases, and 95.42% (*n* = 18,343) of cases were reported as community cases (Suárez et al., 2020). Our results show that the most frequent variants until April 2020 were variants B.1, B.1.609, B.1.243 and B.1.1, which suggests that these variants were already circulating throughout Mexico from the start of the pandemic, and they likely caused the first community cases in Mexico.

Among the 148 variants identified in Mexico, we found five VOCs that have been reported around the world: Alpha, Beta, Gamma, Delta, and Omicron. The Alpha VOC was first detected in the United Kingdom in December 2020 and since then; it has spread to 173 countries, including Mexico (see footnote 1). The first case of the Alpha variant in Mexico confirmed by sequencing was reported on January 10, 2021. Although the isolate was obtained in December 2020 from a 56-year-old British man who lives in Mexico and traveled from Amsterdam to Mexico City on December 28, 2020, and then to Tamaulipas the following day where he was clinically diagnosed.^11^ The Alpha VOC increased in frequency and spread to almost all areas of the Mexican territory. Phylogenetic analysis is in line with epidemiological data, suggesting that this variant arrived in Mexico from Europe. Our analysis suggests that although there have been many introductions from different geographic regions, the Alpha variant that spread throughout Mexico was more similar to the European and United States Alpha isolates than any others.

The Gamma VOC was first identified in Brazil in October 2020 and has spread to 87 countries, mainly in the Americas (see footnote 1). Our phylogenetic analysis suggests that the first introduction of this VOC could have been from South America, Europe, or Asia, although some introductions from Europe and the United States were also detected. The first detected case in Mexico was on January 28, 2021, in Jalisco, a 19-year-old female who had traveled to Brazil and returned to Mexico one day before detection.^12^ This information supports the phylogenetic analysis that the first case was most probably imported from South America. In general, our results suggest that the Gamma VOC population circulating in Mexico was imported from South America, Europe, and the United States, although the viruses from South America were transmitted more than those from other regions.

The Delta VOC was first detected in late 2020 in India and has spread rapidly to 155 countries (see footnote 1). It was first named as the B.1.617 variant, but in May 2021, it was reclassified as Delta (B.1.617.2) and Kappa (B.1.617.1) variants. Mexico reported the first case of the B.1.617 variant in the state of San Luis Potosi in March 2021, in a 40-year-old man who had contact with relatives living in the United States.^13^ The first genome detected as being a Delta VOC (sub-lineage AY.100) was in a 22-year-old woman from Aguascalientes in September 2020, but unfortunately, there was no more information about this patient. These observations suggest that this VOC was introduced into Mexico at least 5 months before the first case was reported in San Luis Potosi. Phylogenetic analyses suggest that the first case of the Delta variant was imported from the United States in September 2020. After that, there were new importations of this VOC from the United States, Asia, and Europe during the first months of 2021, including the first genome identified as lineage B.1.617.2. The three most prevalent Delta sub-lineages in Mexico (AY.20, AY.26 and AY.100) were also highly prevalent in the United States (see footnote 1). In fact, the sub-lineages AY.20 and AY.26 are considered as USA-Mexico lineages due to its high prevalence in both countries; whereas AY.100 is considered as a specific United States lineage.^14^ Introductions from the United States could be explained by the population flow between both countries; Mexico and the United States have the busiest borders crossing in North America. Although transit across this border was restricted during the pandemic, border crossings continued with commercial trade or by people with legal documentation whose movement between the two countries were not restricted.^15^ The states where these three sub-lineages were more prevalent in the United States are also the states with the highest percentage of Mexicans in their population (California, Texas, Arizona, Colorado, New Mexico and Nevada),^16^ and although the pandemic reduced the number of Mexicans returning to Mexico for holidays, there were thousands of Mexicans living in the United States who did visit their relatives in Mexico during 2020-2021.^17^,^18^ In fact, between 2020 and 2021, Mexico registered 4–8 million visitors from the United States.^19^

The Beta variant, which was reported for the first time in South Africa in August 2020 and spread to 119 countries, was the least frequent VOC in Mexico (see footnote 1). The first sequenced genome in Mexico was reported in April 2021; the variant was isolated from a 34-year-old male living in Campeche. Epidemiological information about this VOC in Mexico is limited. Phylogenetic analysis suggests that there were three independent introduction events. In Mexico, its prevalence has been low with the highest level reported in April 2021, but since then, its frequency has dropped and by December 2021, there were no new cases reported (see footnote 3).

The Omicron variant was first reported in South Africa on September 30, 2021, and it has been spreading throughout the world, with 116 countries reporting related cases as of the date of this study period (see footnote 1). In Mexico, this VOC was first reported in a 51-year-old South African man who arrived in Mexico City on November 21, 2021, and he presented COVID-19 symptomatology 6 days later and was diagnosed on November 29, 2021. The presence of the Omicron VOC in Mexico was announced on November 30, 2021.^20^ Nevertheless, the GISAID database reported another Omicron genome isolated in Mexico City on November 16, 2021 (see footnote 3). The patient was a 25-year-old female who traveled to the United States, but unfortunately, there was no more information about this patient. These observations suggest that this VOC was introduced into Mexico, possibly from the United States, up to 1 month before the first case was reported. Phylogenetic analyses indicate that this VOC could have come from the United States, Africa, Europe, and Asia, but the isolates similar to those from the United States, have spread more than those of other regions.

The second most frequent variant in Mexico was the Mexican variant (B.1.1.519), with the first genome being reported in Mexico City on October 13, 2020. Previous studies have determined that this variant derived from variant B.1.1.222, which emerged in Mexico in September 2020 (Lasek-Nesselquist et al., 2021). Interestingly, this variant was reported in other countries from America, Europe, Africa, Asia and Oceania (see footnote 1); however, it does not seem to have spread as efficiently as it did in Mexico. The high frequency of the Mexican variant detected from January to May 2021, and its rapid spread throughout the Mexican territory suggests the need for further investigation in order to determine if this variant was dependent on its adaptation to the Mexican population or to demographic events and understand why some variants are prevalent in specific regions.

Phylogenetic analysis of the Mexican variant showed a comb-like structure in several subclades indicating a rapid exponential growth, which is in line with the rapid increase in its frequency over time, and its demographic dynamics between November 2020 and April 2021. Monophyletic clades and comb-like structures in the VOCs phylogenetic trees were also observed suggesting that these VOCs were maintained through community transmission once it had arrived in Mexico. Epidemiological information from the Mexican Health Ministry in the early stages of the pandemic in Mexico, together with the results from this study, suggest that the pandemic in Mexico was maintained by community transmission, following the importation of variants mainly from the United States, Europe, and South America. This same phenomenon has been reported in other countries in Europe, Asia, Africa and South America (Al Nahid and Ghosh, 2021; Al-Mahruqi et al., 2021; Elizondo et al., 2021; Engelbrecht et al., 2021; Giovanetti et al., 2021; Kostaki et al., 2021; Richter et al., 2021).

The Delta VOC and the Mexican variant were the most prevalent variants reflected in their high effective population sizes (*Ne*). But in general, Alpha, Gamma, Delta, and the Mexican variant presented similar patterns in population dynamics, and after the increase in the *Ne* a stationary phase was reached for each variant. Since *Ne* is related to genetic diversity, more specifically to the nucleotide diversity in each generation (Rouzine et al., 2001), the stabilization of the *Ne* suggests that the same genetic diversity was constant over time. Human interventions such as isolation or quarantine of the population, restricting mobility between countries, and vaccination programs could impact negatively on the *Ne* of the variants. Other studies have reported that the COVID-19 control programs have had a negative impact on the *Ne* of SARS-CoV-2 (Kwon et al., 2021). Interestingly, the Mexican variant presented a strong bottleneck from April 2021 onwards, which coincided with the decline of the number of cases at the end of the second wave, and the emergence and spread of the Alpha, Gamma, and Delta VOCs, which are more transmissible (Thye et al., 2021).

Each variant has several genetic mutations throughout its genome that define them, but the principal mutations that confer biological advantage are located in the Spike protein. The Alpha VOC possesses the N501Y and P681H mutations and more recently, it has been reported that this variant has acquired the E484K mutation (Moustafa et al., 2021), which has been associated with a reduction in the efficacy of both vaccine-induced and natural immunity (Muik et al., 2021; Wang et al., 2021b). The P681H mutation is also involved in reduced antibody recognition (Haynes et al., 2021). Moreover, the N501Y and E484K mutations confer higher transmissibility resulting in rapid spread (PHE, 2020; Davies et al., 2021; Faria et al., 2021; Tanaka et al., 2021). The Gamma and Beta VOCs share the E484K, N501Y and K417T mutations, and several studies suggest that these three mutations cause a significant reduction in viral neutralization by the plasma of convalescent and vaccinated individuals (Cele et al., 2021; de Souza et al., 2021; Greaney et al., 2021; Liu et al., 2021; Wibmer et al., 2021; Wang et al., 2021a). The Delta VOC contains the K417N, E484K, N501Y and P681R/H mutations, as well as the L452R mutation that confers increased infectivity and transmissibility (Deng et al., 2021), escape from antibodies and convalescent sera (Starr et al., 2020; Liu et al., 2021) and the S477N mutation that increases ACE2 binding (Chen et al., 2020; Zahradník et al., 2021), and confers resistance to antibodies and convalescent sera (Gaebler et al., 2021; Liu et al., 2021). Finally, the Omicron VOC harbors the mutations H655Y, N679K and P681H that are associated with increased transmissibility (Gong et al., 2021). It also carries the combination of Q498R and N501Y that increases the binding affinity to ACE2 (Zahradník et al., 2021), as well as other mutations, such as E484K, K417N, L452R and S477N. A recent study demonstrated that the reduction in neutralizing antibodies is compromised by the above mutations, and the requirement of a third vaccine dose is necessary for the effective neutralization of the Omicron VOC (Muik et al., 2021). In Mexico 70% of cases carrying the Omicron VOC occurs in non-vaccinated people.^21^ These observations provide a clear message as to why vaccination is important in the control and prevention of COVID-19.

In summary, the COVID-19 pandemic in Mexico was initiated principally by multiple imported events from Europe and the United States, with subsequent local transmission. Although the Mexican variant (B.1.1.519) was predominant up to May 2021, this was displaced by the subsequent emergence of the Alpha, Gamma, Delta, and Omicron variants. These VOCs are characterized as being more transmissible, and associated with disease severity and immune evasion, all of which represents a threat to public health. It is very important that each country carries out epidemiological and molecular surveillance of the actual VOCs circulating, as well as identifying new variants that carry mutations with biological importance, in order to implement adequate COVID-19 containment programs, as well as encouraging people to get vaccinated.

## Data Availability Statement

All Sequences used in this study are available in the GISAID database https://www.gisaid.org/. Accession numbers can be found in the article/Supplementary Material.

## Author Contributions

AF-A and RM-E contributed to conceptualization. AF-A, GD, LE-C, LS-M and FR-G performed the data curation. AF-A and FR-G performed the methodology and formal analysis. AF-A, LE-C and AC-R contributed to results visualization. AF-A wrote the original draft. GD, LE-C, FR-G, AC-R, LS-M, AC and RM-E reviewed and edited the final manuscript. All authors approved the final version of the manuscript.

## Conflict of Interest

The authors declare that the research was conducted in the absence of any commercial or financial relationships that could be construed as a potential conflict of interest.

## Publisher’s Note

All claims expressed in this article are solely those of the authors and do not necessarily represent those of their affiliated organizations, or those of the publisher, the editors and the reviewers. Any product that may be evaluated in this article, or claim that may be made by its manufacturer, is not guaranteed or endorsed by the publisher.

## Footnotes

^1^ https://outbreak.info/situation-reports

^2^ https://datos.covid-19.conacyt.mx/

^3^ https://www.gisaid.org/

^4^ https://cov-lineages.org/resources/pangolin.html

^5^ https://clades.nextstrain.org/

^6^ https://auspice.us/

^7^ http://tree.bio.ed.ac.uk/software/figtree/

^8^ https://virological.org/t/phylodynamic-analysis-176-genomes-6-mar-2020/356

^9^ http://tree.bio.ed.ac.uk/software/tracer/

^10^ https://www.gob.mx/salud/prensa/077-se-confirma-en-mexico-caso-importado-de-coronavirus-covid-19

^11^ https://www.gob.mx/salud/prensa/009-confirma-secretaria-de-salud-primer-caso-de-nueva-variante-de-sars-cov-2?idiom=es

^12^ https://ssj.jalisco.gob.mx/prensa/noticia/9656

^13^ https://www.capital21.cdmx.gob.mx/noticias/?p=18877

^14^ https://cov-lineages.org/lineage_list.html

^15^ https://nuso.org/articulo/la-frontera-mexico-estados-unidos-asimetrias-y-transgresiones/

^16^ https://en.wikipedia.org/wiki/List_of_U.S._states_by_Hispanic_and_Latino_population

^17^ https://heraldodemexico.com.mx/nacional/2020/12/27/cae-regreso-de-paisanos-mexico-en-temporada-navidena-239244.html

^18^ https://www.dallasnews.com/espanol/al-dia/noticias/2021/12/20/regreso-estados-unidos-mexico-55-mas-paisanos-2021/

^19^ https://www.datatur.sectur.gob.mx/SitePages/VisitantesporNacionalidad.aspx

^20^ https://www.gob.mx/salud/prensa/543-detecta-mexico-la-primera-muestra-positiva-a-la-variante-omicron

^21^ https://presidente.gob.mx/personas-no-vacunadas-las-mas-afectadas-por-contagios-de-covid-19-presidente/
