# Supplementary material for: Two Years of Evolutionary Dynamics of SARS-CoV-2 in Mexico, With Emphasis on the Variants of Concern
Source: Front Microbiol. 2022 Jul 5;13:886585. doi: 10.3389/fmicb.2022.886585 (PMC9294468; doi:10.3389/fmicb.2022.886585)
Supplement: Supplementary file 2 [file Data_Sheet_1.docx]

Supplementary Material

# Supplementary Figures


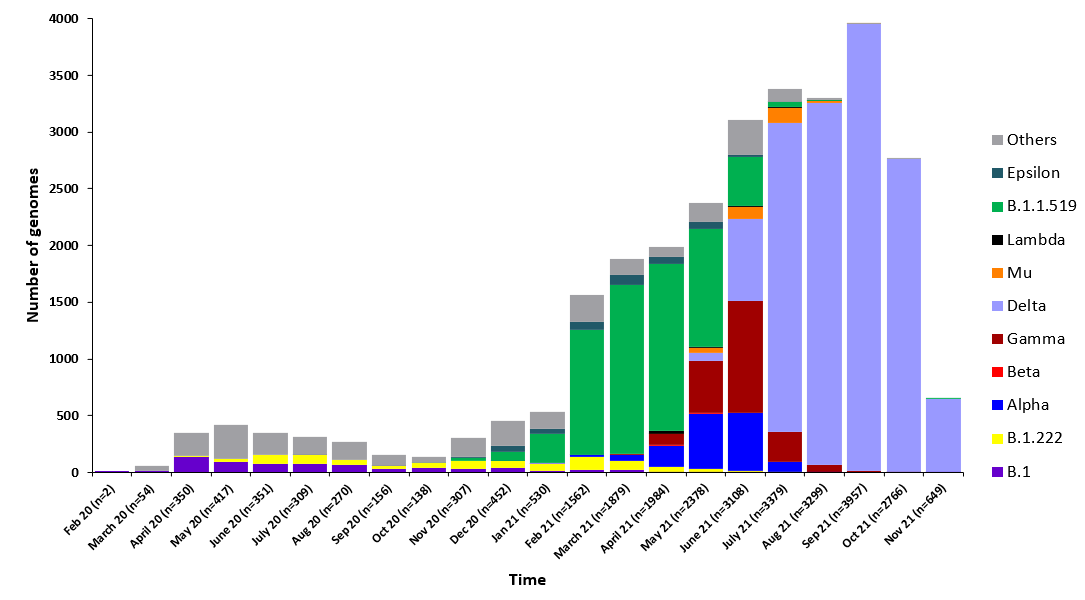
**Supplementary Figure 1.** Breakdown of number of variants of concern (VOCs) and other variants of interest between February 2020 and November 2021 in Mexico. The “Other” category includes variants not classified as VOCs or VOIs with ≤301 genomes sequenced (frequency <1.0 as in Figure 2C).


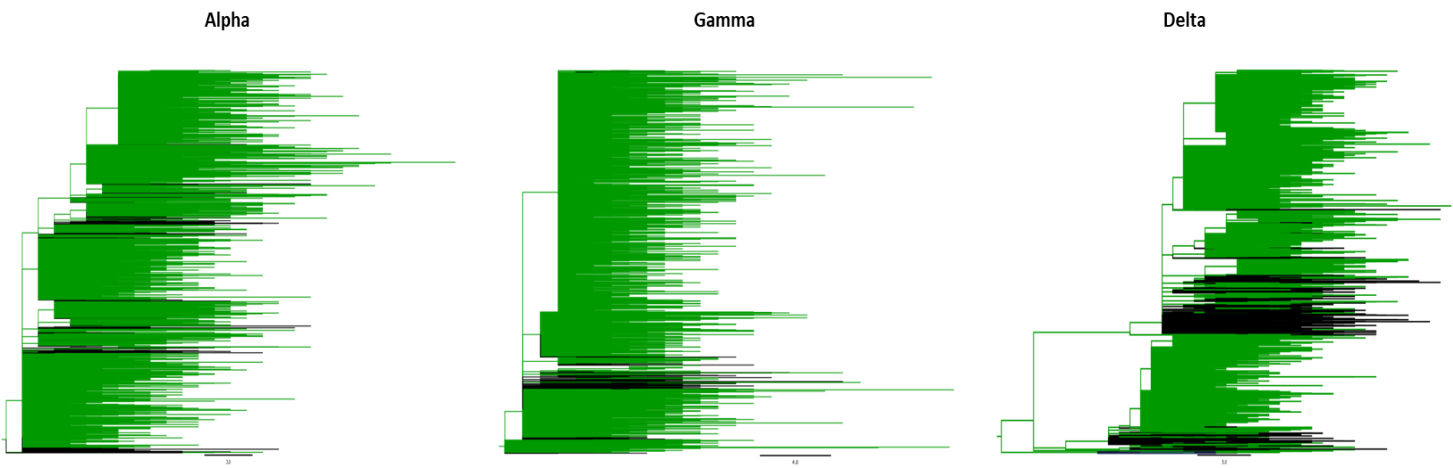


**Supplementary Figure 2.** Phylogenetic trees of Alpha, Gamma and Delta VOCs. The green branches indicate the genomes of Alpha (n = 1370), Gamma (n = 1880) and Delta (n = 5600) isolated in Mexico. The isolates of each variant from other parts of the world that the Nextstrain took as references are shown in black.

**
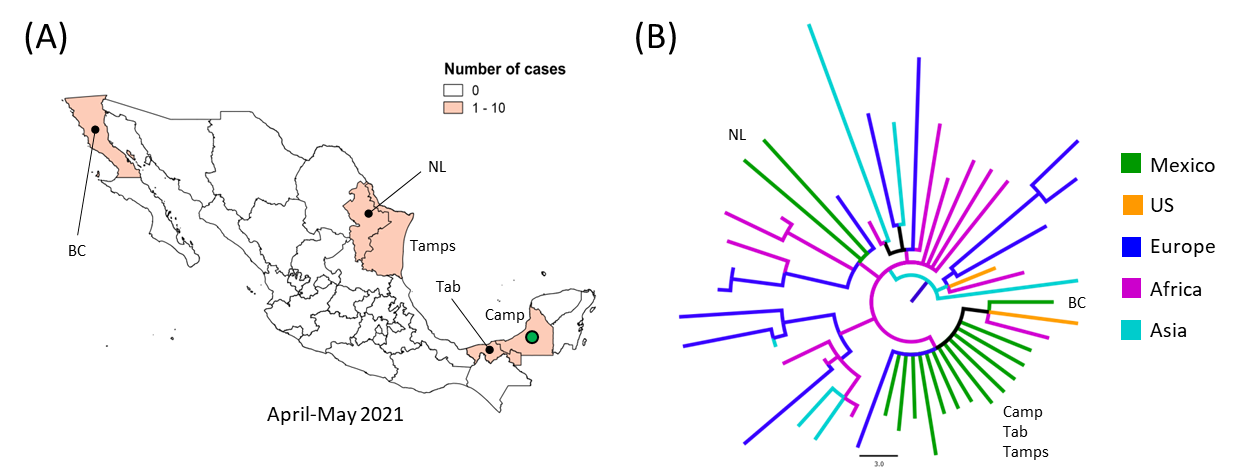
**

**Supplementary Figure 3.** Spatial distribution and phylodynamics of Beta VOC in Mexico. (A) Geographical distribution of the Beta VOC in Mexico. The green circle indicates the Mexican state of Campeche (Camp) where the Beta VOC was first detected. BC, Baja California; NL, Nuevo Leon; Tab, Tabasco; Tamps, Tamaulipas. (B) Phylogenetic analysis of Beta VOC. The green branches indicate the 15 genomes of the Beta VOC isolated in Mexico. The isolates of Beta VOCs taken from other parts of the world that the Nextstrain took as references are shown in other colors. USA, United States of America.

# Supplementary Tables

**Table 1.** Accession numbers, strain, State, collection date, lineage, clade and variant of the 30,645 genomes analyzed.
